# PREPAREDNESS AND CUIDANDO CON RESPETO: A CULTURE-CENTERED APPROACH FOR LATINX CAREGIVERS

**DOI:** 10.1093/geroni/igae098.1707

**Published:** 2024-12-31

**Authors:** Karen Kopera-Frye

**Affiliations:** New Mexico State University, Las Cruces, New Mexico, United States

## Abstract

The Latinx community is the largest racial minority in the United States, yet many Latinx families are in socially vulnerable positions due to immigration crisis, discrimination, health inequities, and poverty. These factors have been shown to impact Latino mental health and well-being; particularly in the caregiving role. The National Academies of Sciences, Engineering, and Medicine (2021) have recommended advancing the field by understanding the cultural aspect of dementia and caregiving in diverse populations and the resultant implications. An Evidence-Based Program, Cuidando con Respeto, aims to empower Spanish-speaking caregivers of loved ones with Alzheimer’s Disease. Fifty-eight Latinx rural dwelling caregivers, primarily daughters, caring for their elder parents with dementia were surveyed about their perceptions of the Cuidando Program and how prepared they felt for their caregiving role. Qualitative analysis of caregiver responses on Program effectiveness and perceptions of preparedness in caregiving areas, e.g., information, revealed overwhelming positive benefits. Caregivers felt more prepared for their caregiving role and appreciated learning coping strategies with disruptive behaviors. The Promotora-led training resulted in greater reported self-efficacy. Level of preparedness is an important caregiving dimension to address, especially with culturally diverse communities having strong cultural caregiving norms. The incidence of Latinx caregivers is rising; however, available culturally appropriate services are lacking, especially in rural areas. Familismo, a Latinx primary cultural value, refers to the importance of strong family loyalty, and contributing to the well-being of the nuclear, and extended family, and kinship networks. Cuidando con Respeto shows promise in empowering caregivers within a familismo-culturally relevant manner.

